# Emotional Eating Under Negative Affect: A Narrative Review from the Perspectives of Emotion Regulation and Reward Processes in Food Choice

**DOI:** 10.3390/nu18111830

**Published:** 2026-06-05

**Authors:** Siwen Fu, Jie Chen, Xiaochun Wang

**Affiliations:** School of Psychology, Shanghai University of Sport, 399 Changhai Road, Yangpu District, Shanghai 200438, China; fusiwen_psy@163.com (S.F.); chenjietrue@163.com (J.C.)

**Keywords:** negative affective states, emotional eating, emotion regulation, reward processing, biased dietary decision-making, nutritional outcomes

## Abstract

Emotional eating under negative affect refers to eating responses that occur in brief unpleasant emotional states and are not explained by hunger alone. This narrative review synthesizes representative evidence from experimental, ecological, and neurocognitive studies on emotional eating under negative affect, with emphasis on two interrelated pathways. (1) Emotion regulation: emotional eating may function as a rapid and accessible regulatory strategy through which food, especially highly palatable food, is used to attenuate negative affect. The immediate soothing effects of eating may reinforce later motivation and habitual responses to regulate emotions through food, whereas more adaptive strategies, such as cognitive reappraisal, may reduce the likelihood and intensity of emotion-related eating. (2) Reward processing and biased decision making: negative affect and affective stress contexts may diminish cognitive control and bias food choice toward immediate rewards. This pathway is reflected in increased attentional bias to food cues, stronger weighting of taste and palatability during value weighing, heightened responsivity to highly rewarding foods, and reduced regulatory influence of health and nutrition attributes. These processes may shift food choice toward energy-dense, nutrient-poor, and ultra-processed foods. The nutritional manifestations of emotional eating are not limited to total intake. Changes in intake quantity are heterogeneous, whereas changes in food choice, diet quality, degree of processing, and eating patterns appear more consistent. Repeated emotional eating may therefore contribute to less stable eating patterns and potential nutritional implications, although links with long-term physiological outcomes remain indirect. Future longitudinal and ecological momentary assessment studies are needed to clarify when emotional eating becomes a stable dietary pattern and which individual or contextual factors increase vulnerability.

## 1. Introduction

An increasing body of research indicates that the associations between diet and nutrient intake and mental health and emotional states are not unidirectional, but rather show a dynamic, bidirectional relationship [1,2]. On the one hand, dietary patterns and nutrient intake may influence individuals’ physical and mental health through pathways such as metabolism and inflammation [3], neurotransmitter synthesis [4], and the gut–brain axis [5]; on the other hand, mental health status and emotional states may, in turn, influence individuals’ dietary choices and eating behaviors [2]. In particular, under contexts of negative affect, individuals may engage in emotional eating for the purpose of alleviating emotions [6]. Emotional eating is most often defined as eating in response to negative emotions rather than hunger alone [7]. This definition does not imply a strict separation between emotional and physiological drivers of eating. Negative affect and stress-related responses may also alter subjective hunger, appetite hormones, and interoceptive signals, suggesting that emotional eating may involve interactions between affective and physiological processes [6]. Although positive emotions may also influence food intake and food choice, there is some debate about whether these responses should be included within the same concept [8]. In a traditional sense, emotional eating primarily refers to eating in response to negative emotions. To clarify the concept of emotional eating and maintain a focused scope, this review limits its discussion to emotional eating under negative affective states. These states are generally brief and shaped by the immediate context, such as sadness, distress, anger, fear, and anxiety [9]. Acute stress is not considered a negative emotion per se; however, acute stress-related contexts are included when they involve negative affective responses or are used to examine momentary dietary decision-making under affective pressure. Evidence from previous studies also suggests that negative emotions may exert a stronger influence on eating behavior. A meta-analysis by Cardi et al. [10] showed that, compared with neutral and positive emotions, laboratory-induced negative emotions were associated with greater food intake. Another ecological momentary assessment (EMA) study found that, compared with time periods with lower negative affect ratings, children tended to choose fried foods with higher fat content during time periods with higher negative affect ratings [11]. These findings suggest that eating under negative affect is not only reflected in changes in quantity but also in changes in food choice. In particular, individuals may be more inclined toward highly rewarding yet less nutrient-dense foods, such as those high in sugar, salt, or fat. When eating is shaped by affective pressure rather than by hunger alone, repeated changes in intake quantity or diet quality may gradually alter the structure of nutrient intake. Over time, these patterns may contribute to metabolic and inflammatory burden and may increase vulnerability to emotional instability and poor mental health.

**Figure 1:**
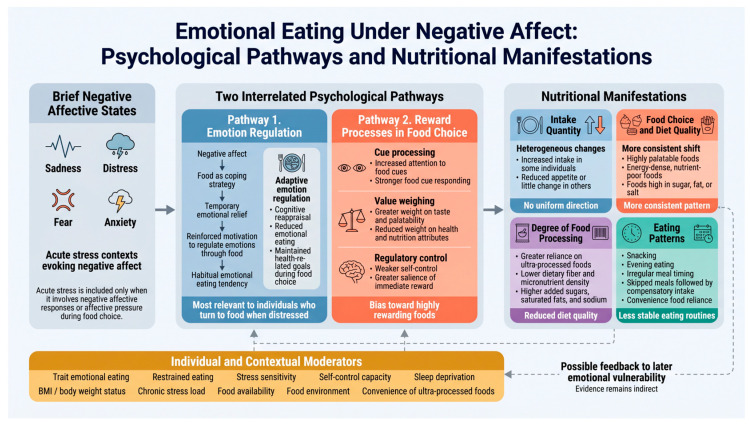
Psychological pathways and nutritional manifestations of emotional eating under negative affect. Solid arrows indicate the proposed conceptual progression from brief negative affective states and affective stress contexts to two interrelated psychological pathways, namely emotion regulation and reward-related processes in food choice, and then to nutritional manifestations. Upward arrows from the moderator box indicate that individual and contextual factors may influence the strength or expression of these pathways. Dashed arrows indicate possible feedback links through which repeated emotional eating and its nutritional manifestations may contribute to later emotional vulnerability. However, these feedback relationships remain indirect and require further longitudinal evidence.

Existing research suggests that changes in eating under contexts of negative affect are often not driven by physiological hunger, but rather resemble a coping and compensatory response. Individuals may regulate or alleviate negative affect through eating (especially highly palatable foods) [10]. At the same time, studies have found that negative affective states may bias individuals toward immediate rewards when making food-related decisions, reduce self-control over food, and thereby influence eating choices [12]. Based on this evidence, the present narrative review examines emotional eating under negative affect, with particular attention to emotion regulation and reward processes involved in food choice [13]. From the perspective of emotion regulation, individuals who experience emotional eating under negative affect may use eating as a rapid and accessible strategy to relieve unpleasant emotional states, and this temporary relief may reinforce later motivation and habitual responses to regulate emotions through food [14]. From the perspective of reward processing and decision bias, negative affect may alter value weighing and self-control during food choice. In this process, sensitivity to immediate food reward cues may increase, whereas the weight placed on longer-term health goals may be reduced. This may make it more difficult to maintain health-oriented food choices and may increase the tendency to choose highly rewarding foods [12]. Using these two pathways as the organizing framework, this review further examines the nutritional consequences of emotional eating under negative affect. Specifically, it considers how emotion regulation and reward processes in food choice may be associated with changes in food intake, diet quality, the degree of food processing, and possible physiological implications. By linking these psychological pathways with nutritional manifestations, this review aims to clarify why emotional eating under negative affect is relevant not only to eating motivation and food choice, but also to broader nutritional and health-related outcomes (The overall mechanistic framework of this review is presented in Figure 1).

As a narrative review, this article synthesizes representative theoretical, experimental, ecological, and review evidence relevant to emotional eating under negative affect, rather than providing an exhaustive systematic review or quantitative synthesis. Within this scope, the present review does not focus on clinical or subclinical affective disorders, such as depressive disorders or anxiety disorders, nor does it treat chronic stress or persistent negative mood as the core exposure. Instead, it focuses on emotional eating in brief negative affective states and on momentary dietary decisions made under affective pressure. In addition, this review does not take clinical eating disorders, including anorexia nervosa, bulimia nervosa, and binge eating disorder, as its primary focus. Although emotional eating may overlap with disordered eating in some individuals, it is treated here as a pattern of affect-related eating rather than as a clinical diagnosis [15].

## 2. Emotion Regulation in Emotional Eating Under Negative Affect

Under negative affect, emotional eating may occur when individuals use food as a coping strategy to regulate unpleasant emotional states [16]. This pathway should be understood as most relevant to people who tend to turn to food when distressed, rather than those who lose appetite or avoid eating under similar conditions. Eating, especially highly palatable food, may provide rapid emotional relief, and this relief may reinforce later motivation and habitual responses to regulate emotions through food. At the same time, the use of emotion regulation strategies may also influence the likelihood and intensity of emotional eating [17]. Adaptive strategies, such as cognitive reappraisal, may reduce emotional eating by helping individuals regulate negative affect and maintain health-related goals during food choice. Together, these two aspects suggest that emotion regulation is relevant not only to the emergence of emotional eating under negative affect, but also to individual differences in how this eating pattern is expressed.

### 2.1. Eating as an Emotion Regulation Strategy Under Negative Affect

In emotional eating under negative affect, unpleasant emotional states may be accompanied by stronger subjective hunger or a stronger urge to eat, even when eating is not driven by physiological hunger alone [18]. Beyond naturalistic contexts, laboratory-induced sadness was followed by stronger subjective hunger, together with increased attentional bias toward food-cue pictures [19], indicating that negative affective states per se can enhance eating motivation. Importantly, this subjective hunger or urge to eat should not be interpreted as purely psychological. Negative affect and stress-related responses may activate the HPA axis and alter appetite-related physiological signals. For example, stress level glucocorticoids have been shown to increase fasting hunger and alter activity in brain regions involved in eating regulation [20]. In psychological stress studies, cortisol responses may also occur together with changes in orexigenic peptides, such as ghrelin, although their effects on eating motivation vary across individuals and contexts [21]. In addition, negative affect is often accompanied by autonomic changes, such as changes in heart rate or gastric sensations [22]. These interoceptive signals may be confused with hunger-related sensations, thereby increasing the urge to eat even when energy need is not the only driver [23].

Emotional eating under negative affect is not entirely explained by changes in physiological or interoceptive signals. The belief that food can alleviate negative emotions may also play a role. Studies have found that negative affect can increase the tendency to cope with unpleasant emotions through eating [24]. Moreover, when individuals expect eating to improve negative affect, negative affect induced by an acute social stress task is more likely to translate into snack intake, particularly among individuals with stronger emotional eating tendencies or greater relief from eating [25]. This evidence suggests that cognitive expectations can, to some extent, strengthen the link from negative affect to eating. From a nutritional perspective, food choices made for emotion regulation often involve foods high in sugar, fat, or salt, which tend to be energy-dense and nutrient-poor [26]. These foods may provide immediate reward, but repeated reliance on them may contribute to higher intakes of free sugars, saturated fats, and sodium, along with lower intakes of dietary fiber and micronutrients [27].

### 2.2. Adaptive Emotion Regulation and Healthier Food Choice

If emotional eating reflects one way of regulating negative affect, then other emotion regulation strategies may influence whether negative affect is translated into food intake or less healthy food choices. Emotion regulation refers to the process by which individuals influence and manage the generation of emotions, the intensity and duration of emotional experiences, and the ways in which emotions are expressed [28]. Cognitive reappraisal and expressive suppression are two commonly used strategies in emotion regulation [29]. O’Leary et al. used a two-study design combining questionnaire assessment and experimental validation to systematically examine the role of cognitive reappraisal in the association between negative affect and food choice [30]. In Study 1, among a sample of university students, cognitive reappraisal tendency, negative affect levels, and dietary patterns were assessed via questionnaires; the results showed that individuals with higher reappraisal tendencies in daily life tended to choose healthier diets, and that negative affect was positively associated with unhealthy eating. In Study 2, negative affect was further induced in an experimental context, and a cognitive reappraisal component was implemented; the results showed that during the negative affect induction phase, individuals reduced the weight placed on the health attribute in food choice. Cognitive reappraisal reduced negative affect and restored the weight of the health attribute to a level close to the midpoint. Together, these two findings indicate that cognitive reappraisal, as an antecedent-focused emotion regulation strategy, can exert a protective effect by adjusting the relative weight assigned to taste and health attributes under negative affect (i.e., increasing the weight of health attributes in decision-making), thereby promoting healthier food choices. By contrast, expressive suppression appears to be less effective in reducing emotional eating under negative affect. One study found that individuals who more habitually used expressive suppression under negative emotions actually ate more [17]. The same study also found that, when negative affect was induced by a negative video (violent clips), participants were directly instructed to use either expressive suppression or cognitive reappraisal while watching, followed by a taste test. The two groups showed comparable levels of negative affect after induction, but the expressive suppression group consumed more highly palatable snack foods during the taste test, some of which may be classified as ultra-processed foods (UPF), such as packaged chocolate products and cakes [17]. These results suggest that negative affect does not inevitably lead to unhealthy emotion-related eating; rather, the key lies in which emotion regulation strategy individuals adopt in the moment to cope. Cognitive reappraisal can maintain or restore the emphasis on “health” in dietary decision-making in the context of negative affect. Expressive suppression may therefore be less helpful for regulating emotional eating and may be associated with greater intake of highly rewarding ultra-processed foods.

Taken together, this section suggests that emotion regulation is relevant to emotional eating under negative affect in two ways. First, negative affect may promote emotional eating through subjective hunger, interoceptive signals, and the expectation that food can alleviate unpleasant emotions. Second, adaptive regulation strategies, especially cognitive reappraisal, may reduce the likelihood that negative affect is translated into less healthy food choices. When emotional eating relies on highly rewarding, nutrient-poor, or ultra-processed foods, repeated episodes may have adverse implications for diet quality. Cognitive reappraisal appears more likely to support healthier choices, whereas expressive suppression may be associated with greater intake of highly rewarding ultra-processed foods.

## 3. Reward Processes and Biased Food Choice Under Negative Affect

In emotional eating under negative affect, food choice may be shaped not only by the use of eating as a way to regulate unpleasant emotions, but also by changes in reward processing, inhibitory control, and goal maintenance [31]. Negative affect, as well as acute stress paradigms that evoke negative affective responses, may increase the salience of immediate food reward while reducing the influence of longer-term health goals [32]. Therefore, even when individuals cognitively recognize the potential costs of unhealthy eating, they may still find it more difficult to inhibit immediate approach responses toward highly rewarding foods, manifested as an increased tendency to choose foods high in sugar/fat/salt, with energy-dense, nutrient-poor characteristics. Existing evidence supports this pathway at three related levels: cue processing, value weighing during food choice, and neural mechanisms. (1) Cue-processing level. Studies have shown that negative emotion, compared with neutral emotion, can increase attentional bias toward food cues, and this attentional bias is positively associated with subjective appetite [19]. Studies using acute social stress paradigms have also found stronger implicit wanting for sweet and high-fat food pictures [33]. (2) Value weighing during food choice. Regarding value weighing during food choice, individuals may integrate several food attributes, such as taste, health, and nutrition, into a subjective value signal [34]. Studies have found that, under acute stress, individuals may place greater weight on taste attributes during food choice [12]. (3) Neural mechanism level: enhanced responses to highly rewarding food cues and weakened regulation of health attributes. To further explain why negative affect/acute stress drives changes in eating (e.g., dysregulated quantity; shifts toward foods high in sugar/fat/salt, with energy-dense, nutrient-poor characteristics); behavioral findings alone are insufficient to reveal the underlying processing mechanisms. Therefore, this level adds evidence from neural mechanisms to complement the behavioral and measurement-based findings described above. One study used functional magnetic resonance imaging (fMRI) to examine brain activity in 30 chronic dieters while they viewed tempting food pictures after negative versus neutral emotion induction. Compared with the neutral emotion condition, negative emotion induction was associated with a marked increase in orbitofrontal cortex activity when participants viewed tempting foods. This finding suggests that negative emotion may heighten reward-related responses to appetitive food cues and supports the association between negative emotion and disinhibited eating [32]. During food choice, individuals often weigh taste against health [35]. Hare et al. showed that the ventromedial prefrontal cortex integrates attributes such as taste and health into a subjective value signal, whereas prefrontal control regions, including the dorsolateral prefrontal cortex, help incorporate health information into this valuation process [36]. This evidence suggests that maintaining health goals during food choice depends partly on the ability of control networks to modulate value computation. Under negative affect or acute stress paradigms that evoke affective pressure, inhibitory control may decline. Acute stress or intense negative emotions can rapidly weaken prefrontal executive control-related functions [37]. In food self-control choice contexts, this influence is not only reflected in reduced behavioral self-control but is also manifested as changes in functional connectivity at key nodes of the decision-making circuitry. For example, one acute stress study found that participants showed reduced self-control in food choice, together with weaker functional connectivity between prefrontal control regions and regions involved in value computation [12]. These findings suggest that stress may reduce the influence of control networks on value computation, making it more difficult for health and nutrition attributes to shape dietary decisions. From a nutritional perspective, this pathway suggests that emotional eating under negative affect may shift food choice toward taste and immediate reward, while reducing attention to nutrient density, energy density, and longer-term health consequences. Such shifts may increase preference for energy-dense, nutrient-poor foods, especially foods high in sugar, fat, or salt.

Taken together, these findings indicate that reward processes in food choice may help explain why emotional eating under negative affect often involves stronger food cue responding, greater weight on immediate reward, and reduced influence of health-related goals.

## 4. Nutritional Manifestations and Potential Implications of Emotional Eating Under Negative Affect

The nutritional relevance of emotional eating under negative affect lies not only in how much individuals eat, but also in what they choose to eat and how these eating responses are organized over time. Changes in intake quantity are often heterogeneous, whereas changes in diet quality and food processing may show more consistent shifts toward highly palatable, energy-dense, and nutrient-poor foods. These dietary manifestations provide a basis for considering the possible nutritional and health-related implications of emotional eating under negative affect.

### 4.1. Intake Quantity and Individual Differences

Although this review focuses on emotional eating as a food approach response, intake quantity under negative affect should not be reduced to overeating alone. Some individuals may show increased intake, whereas others may show reduced appetite or little change in total intake. For example, the same stress context may increase intake, but may also lead to decreased appetite and reduced intake [38]. Under negative affect, changes in intake amount likewise show directional divergence, and the average trend more often involves decreased appetite or unchanged intake. That is, negative affect does not inevitably lead to eating more, but depends on whether individuals possess vulnerability characteristics such as a stronger tendency toward emotion-related eating. One review-based line of evidence indicated that, in most cases, negative affect is more consistently associated with reduced intake, whereas increased intake tends to be concentrated in specific subgroups (e.g., individuals high in emotional eating, and some restrained eaters) [39]. A behavioral study similarly showed that, after negative affect was induced, individuals high in emotional eating were more likely to exhibit increased intake, thereby explaining why overall findings show pronounced individualized differences [40]. In addition, factors such as a history of restrained eating [41], Body Mass Index (BMI) level [42], sleep deprivation [43], and chronic stress load [44] may also determine whether individuals under negative affect are more likely to display an “increased intake” pattern or a “decreased intake” pattern. These factors may influence intake responses in different ways. Restrained eating may increase vulnerability to disinhibited eating when negative affect weakens dietary restraint [41]. Body weight status may moderate the association between stress and emotional eating, with stronger stress-related eating observed in some higher BMI samples [42]. Sleep deprivation may increase snack intake by reducing self-control and increasing reward sensitivity to palatable foods [43]. Chronic stress load may further strengthen habitual emotion-related eating and increase reliance on convenient, highly palatable foods [44].

Marked increases or decreases in intake may have nutritional relevance when they occur repeatedly, especially if they disrupt regular energy intake, meal timing, or dietary balance. First, changes in eating under negative affect/acute stress can themselves be viewed as part of an emotion-induced physiological stress response. Evidence indicates that negative affect and acute stress can activate the HPA axis and the autonomic nervous system, thereby triggering a series of physiological and behavioral changes, including changes in eating behavior [45,46]. Rather than treating changes in intake quantity as direct evidence of pathophysiological impairment, this review discusses them as dietary manifestations of emotional eating. Repeated increases in intake may raise total energy intake, especially when the additional foods are snacks, sweets, or energy-dense, nutrient-poor foods. Repeated decreases in intake may also be nutritionally relevant when they involve skipped meals, irregular meal timing, or reduced intake of nutrient-rich foods. Therefore, the main nutritional issue is whether affect-related increases or decreases in intake become recurrent patterns that alter energy distribution, meal regularity, and nutrient adequacy. In addition, short-term energy surplus may disrupt the dynamic balance of appetite-regulating hormones [47], and strengthen the emotion–eating link via the reward system, thereby increasing the risk of subsequent eating instability and repeated excessive intake [48]. Finally, when food intake quantity shows an abnormal decrease in the short term, it can likewise produce adverse effects on human functioning. On the one hand, short-term insufficient energy intake may lead to inadequate glucose supply, thereby affecting attention, executive function, and emotional stability, and increasing the risk of fatigue and reduced cognitive efficiency [49]. On the other hand, persistent or recurrent low energy intake may reduce energy availability, thereby affecting endocrine regulation and metabolic homeostasis, and to some extent disrupting the normal feedback mechanisms of appetite-related hormones [47]. In addition, irregular eating or frequently skipping regular meals has also been considered to be associated with adverse metabolic outcomes, including difficulties in weight regulation and increased risk of metabolic syndrome [50]. Therefore, even short-term reduced intake, in the context of repeated emotional fluctuations, may become a starting point for the accumulation of long-term nutritional imbalance and metabolic risk.

Taken together, the evidence suggests that intake quantity under negative affect is heterogeneous, whereas diet quality may show a more consistent shift. This heterogeneity in intake quantity makes it necessary to examine diet quality and food processing as more stable nutritional manifestations of emotional eating.

### 4.2. Diet Quality and Processed Food Choices in Emotional Eating

Compared with intake quantity, diet quality appears to show a more consistent pattern in emotional eating under negative affect. When individuals are under negative emotions or acute stress, compared with the divergent changes in intake quantity, changes in the “quality” of food choices show a more consistent trend. A review-based study indicated that, in negative affect contexts, individuals tend to choose foods with higher energy density and higher fat or sugar content, whereas choices of healthy foods (e.g., vegetables, fruits, and whole grains) are relatively reduced [51]. This choice tendency is widely referred to as the “comfort food” phenomenon. Meanwhile, both experimental and observational studies have shown a stable association between negative affective states and increased intake of sweets and high-fat foods, because such foods typically have higher sensory pleasantness and features of immediate gratification [52,53].

This phenomenon mainly occurs for two reasons. On the one hand, high-sugar and high-fat foods are highly attractive and can provide individuals with pleasurable experiences and a subjective sense of “comfort” in a short time, and thus are more likely to be chosen under low mood or stress contexts [52]. On the other hand, when individuals are under time pressure or in contexts of limited psychological resources, they may be more inclined to choose UPF with high convenience [54]. However, from a nutritional perspective, a shift in food “quality” under negative affect does not only mean increased intake of high-fat and high-sugar foods; more importantly, it indicates changes in the structure of dietary nutrients. High-energy-dense and UPF typically have nutritional characteristics of “high energy density, high levels of added sugars and saturated fats, and relatively low density of dietary fiber, vitamins, minerals, and other micronutrients” [55]. In this context, even if total energy intake does not increase significantly, reduced nutrient density in dietary structure may still weaken the body’s capacity for metabolic regulation. First, at the metabolic level, diets high in energy density and added sugars are more likely to induce postprandial fluctuations in blood glucose and blood lipids, accompanied by enhanced low-grade inflammatory responses [56]. In the long term, repeated occurrence of such postprandial metabolic loads is considered an important early phenotype of cardiometabolic disease. Second, UPF often lack dietary fiber and the nutritional structure of natural foods, and their rapid digestion and absorption properties may weaken the persistence of satiety signals and increase subsequent eating instability [55,57]. The gut microbiota may also be relevant to this process. Gut microbial metabolites and gut-derived hormones are involved in appetite regulation and gut–brain communication, and stress may disturb microbiota composition and gut barrier function [58]. Therefore, repeated emotional eating patterns characterized by low fiber intake and greater reliance on ultra-processed foods may have potential implications for appetite regulation through the microbiota gut–brain axis. However, this pathway remains indirect in the context of emotional eating and should be examined more directly in future studies. In addition, higher proportions of added sugars and highly refined carbohydrates are also closely associated with increased risk of insulin resistance [59]. At the epidemiological level, multiple large prospective cohort studies and systematic reviews have consistently found that a higher proportion of ultra-processed food intake is significantly associated with increased risks of obesity, metabolic syndrome, type 2 diabetes, and cardiovascular disease. For example, a French cohort study showed that for each 10% increase in the proportion of intake, the risk of cardiovascular disease increased significantly [60]. Similar findings have been repeatedly confirmed in other multinational cohort studies [61,62]. Therefore, a preference for high-energy-dense and UPF in the context of negative affect, even if it does not immediately manifest as weight gain, may gradually contribute to the long-term development of cardiometabolic risk through reduced diet quality, imbalanced nutrient structure, and accumulated metabolic load. However, it should be noted that these longer-term risks should be interpreted as potential implications inferred from broader evidence on diet quality and ultra-processed food intake, rather than as direct evidence that emotional eating alone causes cardiometabolic disease.

Thus, diet quality and food processing may represent more consistent nutritional manifestations of emotional eating than total intake quantity. When such choices recur across emotional contexts, they may contribute to less stable eating patterns over time.

### 4.3. Repeated Emotional Eating and Dietary Pattern Instability

When emotional eating recurs across similar negative affective contexts, it may gradually become a more habitual way of responding to distress. On the one hand, although emotion-related eating can be effective as a coping strategy for short-term relief of negative affect, this experience itself does not truly alleviate emotional distress and may instead gradually become a dependent behavior. Long-term, frequent reliance on high-energy-dense, high-fat, and high-sugar foods to cope with emotions may lead this behavior to be regarded as the default coping strategy under negative affect, thereby forming a habitual pattern. For example, an animal study found that under repeated chronic stress conditions, rats consumed more comfort food, and this intake also reduced HPA axis responses during subsequent stress, forming a feedback chain of “using palatable food to relieve stress,” which provides experimental evidence for the consolidation pathway of emotion-related eating [63]. At the same time, a behavioral study showed that after a designed laboratory stress task, individuals who reported higher emotional eating and obtained stronger emotional relief from eating were more likely to increase snack intake after stress [64]. Repeated emotional eating may also be expressed through eating patterns rather than intake quantity alone. Some individuals may show more frequent snacking or evening eating, whereas others may show irregular meal timing, skipped meals followed by compensatory intake, or a shift toward convenient ultra-processed foods during periods of distress. These patterns are nutritionally relevant because they may affect total energy intake, nutrient distribution across the day, and the stability of appetite regulation. The accumulation of this behavioral pattern over time not only increases total energy intake, but also gradually strengthens the link between eating and emotional experiences, ultimately leading food choice to be increasingly driven by negative affect rather than by physiological hunger. On the other hand, the cumulative effects of negative-affect-driven dietary patterns are also reflected at the metabolic level. Long-term unhealthy dietary patterns, particularly repeated intake of high-calorie and highly processed foods, are closely associated with abnormal weight gain, dysregulated fat metabolism, and insulin resistance. These phenomena have been observed in longitudinal studies and linked to emotion-related eating behaviors [65]. Negative affect or stress states are often accompanied by sustained activation of the HPA axis and elevated cortisol levels. This endocrine response not only promotes individuals’ preference for high-energy foods, but may also lead to fat accumulation and remodeling of energy metabolism, thereby increasing the risk of obesity, metabolic syndrome, and cardiovascular-related diseases [66].

Compared with the time-accumulated effects of negative-affect-driven dietary patterns discussed above, a more complex and noteworthy issue is that a vicious bidirectional cycle may form between dietary patterns and negative affective states. In the short term, individuals may obtain temporary emotional relief through emotion-related eating; however, the relief effect is transient and unstable. In the long term, an adverse metabolic state itself may, in turn, impair individuals’ emotion regulation capacity and exacerbate negative affective experiences, thereby making individuals more likely to choose comfort food again in subsequent similar contexts [67]. At the subjective level, some individuals may experience a repeated pattern in which eating brings short-term relief, followed by regret or worsened mood, which may increase the likelihood of turning to comfort food again in similar emotional contexts [68]. The association between long-term, emotion-related eating and adverse metabolic factors has also been discussed as one possible pathway linking dietary patterns with broader health-related risk [14]. However, this feedback pathway should be interpreted cautiously, because current evidence does not establish that emotional eating alone directly causes long-term metabolic or psychological outcomes. Future longitudinal and ecological momentary assessment studies are needed to clarify when repeated emotional eating becomes a stable dietary pattern and how it relates to later nutritional and emotional outcomes.

Overall, repeated emotional eating under negative affect may contribute to habitual reliance on highly rewarding foods and to less stable eating patterns. However, the evidence linking short-term emotional eating to long-term physiological outcomes remains indirect. Future longitudinal and ecological momentary assessment studies are needed to clarify when these repeated responses develop into stable dietary patterns and how they relate to later nutritional and health-related outcomes.

## 5. Limitations and Future Directions

Several limitations should be considered when interpreting the present review. First, this article adopts a narrative approach and synthesizes representative evidence rather than providing an exhaustive systematic review or quantitative synthesis. This approach is appropriate for clarifying concepts and integrating theoretical, behavioral, nutritional, and neurocognitive findings, but it does not allow conclusions about pooled effect sizes or the relative strength of different pathways [69]. Because the reviewed studies differ in sample size, age and sex composition, stress or emotion induction procedures, food types, observation windows, and dietary assessment methods, this narrative review does not attempt to provide pooled percentages or uniform estimates of intake change. Future systematic reviews and meta-analyses should quantify these effects where sufficient comparable data are available. Future reviews could complement the present synthesis with more systematic search strategies and, where the evidence base allows, quantitative analyses comparing the effects of negative affect on intake quantity, diet quality, and food choice. Second, emotional eating remains a heterogeneous construct. In this review, the concept was used in its traditional and narrower sense, referring mainly to eating in response to negative affect. This focus helped maintain a clear scope, but it also means that eating in response to positive emotions was not discussed in detail. Future work should further distinguish negative emotional eating from positive emotional eating, because these forms of eating may involve different motives, food choices, and health-related implications [70]. Third, the available evidence varies substantially in measurement approach and ecological validity. Some studies rely on self-report questionnaires, whereas others use laboratory mood induction, acute stress paradigms, taste tests, ecological momentary assessment, or neuroimaging tasks. These methods capture different aspects of emotional eating, and self-reported emotional eating may not always correspond to actual food intake under negative affect [8]. Future studies should combine self-report, objective intake measures, ecological momentary assessment, and, where appropriate, physiological or neural indicators [6]. Such multi-method designs would help clarify when negative affect leads to food approach, when it leads to reduced appetite, and when it mainly alters food choice without changing total intake. Fourth, the boundary conditions of emotional eating under negative affect remain insufficiently specified. The pathways reviewed here are most relevant to individuals who tend to turn to food when distressed, but this response may depend on traits such as emotional eating, restrained eating, stress sensitivity, sleep, body weight status, food availability, and broader social and environmental contexts. Future research should move beyond average effects and examine individual patterns over time. Person-specific and longitudinal designs may be especially useful for identifying who is most likely to show reward-based food choices, increased snack intake, irregular eating patterns, or reliance on ultra-processed foods during periods of negative affect. Finally, the nutritional and physiological implications discussed in this review should be interpreted cautiously. Evidence more consistently supports changes in food choice and diet quality than changes in total intake quantity. Moreover, links between repeated emotional eating, ultra-processed food intake, metabolic disturbance, and later emotional vulnerability are still largely indirect. Future studies should examine whether repeated emotional eating prospectively predicts stable dietary patterns, nutrient intake profiles, and health-related outcomes [71]. Longitudinal studies with repeated measures of affect, food choice, dietary intake, and metabolic markers would be particularly valuable for testing whether emotional eating under negative affect develops into a persistent pattern with broader nutritional consequences.

## 6. Conclusions

This narrative review focused on emotional eating under negative affect and examined how this phenomenon can be understood through two interrelated psychological pathways: emotion regulation and reward processes in food choice. From the perspective of emotion regulation, emotional eating may function as a coping response in which food, especially highly palatable food, is used to soften unpleasant emotional states. The temporary relief obtained from eating may strengthen later motivation to regulate emotions through food and may gradually reinforce habitual emotional eating responses. At the same time, adaptive emotion regulation strategies, particularly cognitive reappraisal, may help reduce negative affect and maintain attention to health-related goals during food choice. From the perspective of reward processes in food choice, negative affect may increase sensitivity to immediate food reward cues and alter the relative weight assigned to taste, reward, health, and nutrition attributes. Evidence from cue processing, value weighing, and neurocognitive studies suggests that negative affect and affective stress contexts may make highly rewarding foods more salient, weaken self-control during food choice, and reduce the influence of longer-term health considerations. The nutritional relevance of emotional eating under negative affect is reflected not only in food intake quantity, but also in diet quality, degree of food processing, and eating patterns. Changes in total intake are heterogeneous: some individuals may eat more, whereas others may show reduced appetite or little change. By contrast, shifts in food choice and diet quality appear more consistent, particularly in the direction of highly palatable, energy-dense, nutrient-poor, and ultra-processed foods. When emotional eating recurs across similar affective contexts, it may also contribute to less stable eating patterns, such as more frequent snacking, evening eating, irregular meal timing, or greater reliance on convenient processed foods. These manifestations suggest that emotional eating under negative affect has implications beyond momentary eating motivation and may gradually shape dietary structure and broader nutritional health. Overall, this review links emotion regulation and reward-based food choice to the nutritional manifestations of emotional eating under negative affect. Future research should use longitudinal and ecological momentary assessment designs to clarify when emotional eating becomes a stable dietary pattern, how it affects nutrient intake and diet quality over time, and which individual or contextual factors increase vulnerability to this pattern.

## Data Availability

No new data were created or analyzed in this study. Data sharing is not applicable to this article.

## Abbreviations

The following abbreviations are used in this manuscript:

EMA	Ecological Momentary Assessment Study
HPA	Hypothalamic–Pituitary–Adrenal
UPF	ultra-processed foods
vmPFC	ventromedial prefrontal cortex
DLPFC	dorsolateral prefrontal cortex
BMI	Body Mass Index

## References

[1] Polivy J., Herman C.P. (2005). Mental Health and Eating Behaviours: A Bi-Directional Relation. Can. J. Public Health.

[2] van der Pols J.C. (2018). Nutrition and mental health: Bidirectional associations and multidimensional measures. Public Health Nutr..

[3] Wang J., Zhou Y., Chen K., Jing Y., He J., Sun H., Hu X. (2019). Dietary Inflammatory Index and Depression: A Meta-Analysis. Public Health Nutr..

[4] Wurtman R.J., Wurtman J.J., Regan M.M., McDermott J.M., Tsay R.H., Breu J.J. (2003). Effects of Normal Meals Rich in Carbohydrates or Proteins on Plasma Tryptophan and Tyrosine Ratios. Am. J. Clin. Nutr..

[5] Berding K., Vlckova K., Marx W., Schellekens H., Stanton C., Clarke G., Jacka F., Dinan T.G., Cryan J.F. (2021). Diet and the Microbiota–Gut–Brain Axis: Sowing the Seeds of Good Mental Health. Adv. Nutr..

[6] Reichenberger J., Schnepper R., Arend A.-K., Blechert J. (2020). Emotional Eating in Healthy Individuals and Patients with an Eating Disorder: Evidence from Psychometric, Experimental and Naturalistic Studies. Proc. Nutr. Soc..

[7] Arnow B., Kenardy J., Agras W.S. (1995). The Emotional Eating Scale: The Development of a Measure to Assess Coping with Negative Affect by Eating. Int. J. Eat. Disord..

[8] Bongers P., Jansen A. (2016). Emotional Eating Is Not What You Think It Is and Emotional Eating Scales Do Not Measure What You Think They Measure. Front. Psychol..

[9] Zheng J., Li S., Wang T., Lajoie S.P. (2024). Unveiling Emotion Dynamics in Problem-Solving: A Comprehensive Analysis with an Intelligent Tutoring System Using Facial Expressions and Electrodermal Activities. Int. J. Educ. Technol. High. Educ..

[10] Cardi V., Leppanen J., Treasure J. (2015). The Effects of Negative and Positive Mood Induction on Eating Behaviour: A Meta-Analysis of Laboratory Studies in the Healthy Population and Eating and Weight Disorders. Neurosci. Biobehav. Rev..

[11] Naya C.H., Chu D., Wang W.-L., Nicolo M., Dunton G.F., Mason T.B. (2022). Children’s Daily Negative Affect Patterns and Food Consumption on Weekends: An Ecological Momentary Assessment Study. J. Nutr. Educ. Behav..

[12] Maier S.U., Makwana A.B., Hare T.A. (2015). Acute Stress Impairs Self-Control in Goal-Directed Choice by Altering Multiple Functional Connections within the Brain’s Decision Circuits. Neuron.

[13] Macht M. (2008). How Emotions Affect Eating: A Five-Way Model. Appetite.

[14] Dakanalis A., Mentzelou M., Papadopoulou S.K., Papandreou D., Spanoudaki M., Vasios G.K., Pavlidou E., Mantzorou M., Giaginis C. (2023). The Association of Emotional Eating with Overweight/Obesity, Depression, Anxiety/Stress, and Dietary Patterns: A Review of the Current Clinical Evidence. Nutrients.

[15] American Psychiatric Association (APA) (2013). Diagnostic and Statistical Manual of Mental Disorders: DSM-5^TM^.

[16] Spoor S.T., Bekker M.H., Van Strien T., van Heck G.L. (2007). Relations between Negative Affect, Coping, and Emotional Eating. Appetite.

[17] Evers C., Marijn Stok F., de Ridder D.T.D. (2010). Feeding Your Feelings: Emotion Regulation Strategies and Emotional Eating. Pers. Soc. Psychol. Bull..

[18] Huh J., Shiyko M., Keller S., Dunton G., Schembre S.M. (2015). The Time-Varying Association between Perceived Stress and Hunger within and between Days. Appetite.

[19] Hepworth R., Mogg K., Brignell C., Bradley B.P. (2010). Negative Mood Increases Selective Attention to Food Cues and Subjective Appetite. Appetite.

[20] Bini J., Parikh L., Lacadie C., Hwang J.J., Shah S., Rosenberg S.B., Seo D., Lam K., Hamza M., De Aguiar R.B. (2022). Stress-Level Glucocorticoids Increase Fasting Hunger and Decrease Cerebral Blood Flow in Regions Regulating Eating. NeuroImage Clin..

[21] Rouach V., Bloch M., Rosenberg N., Gilad S., Limor R., Stern N., Greenman Y. (2007). The Acute Ghrelin Response to a Psychological Stress Challenge Does Not Predict the Post-Stress Urge to Eat. Psychoneuroendocrinology.

[22] Kreibig S.D. (2010). Autonomic Nervous System Activity in Emotion: A Review. Biol. Psychol..

[23] Herbert B.M., Pollatos O. (2012). The Body in the Mind: On the Relationship Between Interoception and Embodiment. Top. Cogn. Sci..

[24] Macht M., Simons G. (2000). Emotions and Eating in Everyday Life. Appetite.

[25] Klatzkin R.R., Nadel T., Wilkinson L.L., Gaffney K., Files H., Gray Z.J., Slavich G.M. (2023). Lifetime Stressor Exposure, Eating Expectancy, and Acute Social Stress-Related Eating Behavior: A Pre-Registered Study of the Emotional Eating Cycle. Appetite.

[26] Camilleri G.M., Méjean C., Kesse-Guyot E., Andreeva V.A., Bellisle F., Hercberg S., Péneau S. (2014). The Associations between Emotional Eating and Consumption of Energy-Dense Snack Foods Are Modified by Sex and Depressive Symptomatology. J. Nutr..

[27] Monteiro C.A., Cannon G., Moubarac J.-C., Levy R.B., Louzada M.L.C., Jaime P.C. (2018). The UN Decade of Nutrition, the NOVA Food Classification and the Trouble with Ultra-Processing. Public Health Nutr..

[28] Gross J.J. (1998). The Emerging Field of Emotion Regulation: An Integrative Review. Rev. Gen. Psychol..

[29] Gross J.J., John O.P. (2003). Individual Differences in Two Emotion Regulation Processes: Implications for Affect, Relationships, and Well-Being. J. Pers. Soc. Psychol..

[30] O’Leary D., Smith A., Salehi E., Gross J.J. (2023). Negative Affect, Affect Regulation, and Food Choice: A Value-Based Decision-Making Analysis. Soc. Psychol. Personal. Sci..

[31] Byrne M.E., Shank L.M., Altman D.R., Swanson T.N., Ramirez E., Moore N.A., Rubin S.G., LeMay-Russell S., Parker M.N., Kaufman R.E. (2021). Inhibitory Control and Negative Affect in Relation to Food Intake among Youth. Appetite.

[32] Wagner D.D., Boswell R.G., Kelley W.M., Heatherton T.F. (2012). Inducing Negative Affect Increases the Reward Value of Appetizing Foods in Dieters. J. Cogn. Neurosci..

[33] Hyldelund N.B., Dalgaard V.L., Byrne D.V., Andersen B.V. (2022). Why Being ‘Stressed’ Is ‘Desserts’ in Reverse—The Effect of Acute Psychosocial Stress on Food Pleasure and Food Choice. Foods.

[34] Rangel A. (2013). Regulation of Dietary Choice by the Decision-Making Circuitry. Nat. Neurosci..

[35] Hare T.A., Malmaud J., Rangel A. (2011). Focusing Attention on the Health Aspects of Foods Changes Value Signals in vmPFC and Improves Dietary Choice. J. Neurosci..

[36] Hare T.A., Camerer C.F., Rangel A. (2009). Self-Control in Decision-Making Involves Modulation of the vmPFC Valuation System. Science.

[37] Pessoa L. (2009). How Do Emotion and Motivation Direct Executive Control?. Trends Cogn. Sci..

[38] Hill D., Conner M., Clancy F., Moss R., Wilding S., Bristow M., O’Connor D.B. (2022). Stress and Eating Behaviours in Healthy Adults: A Systematic Review and Meta-Analysis. Health Psychol. Rev..

[39] Ha O.-R., Lim S.-L. (2023). The Role of Emotion in Eating Behavior and Decisions. Front. Psychol..

[40] van Strien T., Cebolla A., Etchemendy E., Gutiérrez-Maldonado J., Ferrer-García M., Botella C., Baños R. (2013). Emotional Eating and Food Intake after Sadness and Joy. Appetite.

[41] Yeomans M.R., Coughlan E. (2009). Mood-Induced Eating. Interactive Effects of Restraint and Tendency to Overeat. Appetite.

[42] Nguyen-Rodriguez S.T., Chou C.-P., Unger J.B., Spruijt-Metz D. (2008). BMI as a Moderator of Perceived Stress and Emotional Eating in Adolescents. Eat. Behav..

[43] Dweck J.S., Jenkins S.M., Nolan L.J. (2014). The Role of Emotional Eating and Stress in the Influence of Short Sleep on Food Consumption. Appetite.

[44] Tuluhong M., Han P. (2023). Chronic Stress Is Associated with Reward and Emotion-Related Eating Behaviors in College Students. Front. Nutr..

[45] Torres S.J., Nowson C.A. (2007). Relationship between Stress, Eating Behavior, and Obesity. Nutrition.

[46] Ulrich-Lai Y.M., Herman J.P. (2009). Neural Regulation of Endocrine and Autonomic Stress Responses. Nat. Rev. Neurosci..

[47] Sumithran P., Prendergast L.A., Delbridge E., Purcell K., Shulkes A., Kriketos A., Proietto J. (2011). Long-Term Persistence of Hormonal Adaptations to Weight Loss. N. Engl. J. Med..

[48] Volkow N.D., Wang G.-J., Tomasi D., Baler R.D. (2013). Obesity and Addiction: Neurobiological Overlaps. Obes. Rev..

[49] Chang C.-Y., Ke D.-S., Chen J.-Y. (2009). Essential Fatty Acids and Human Brain. Acta Neurol. Taiwanica.

[50] St-Onge M.-P., Ard J., Baskin M.L., Chiuve S.E., Johnson H.M., Kris-Etherton P., Varady K., American Heart Association Obesity Committee of the Council on Lifestyle and Cardiometabolic Health, Council on Cardiovascular Disease in the Young, Council on Clinical Cardiology (2017). Meal Timing and Frequency: Implications for Cardiovascular Disease Prevention: A Scientific Statement From the American Heart Association. Circulation.

[51] Evers C., Dingemans A., Junghans A.F., Boevé A. (2018). Feeling Bad or Feeling Good, Does Emotion Affect Your Consumption of Food? A Meta-Analysis of the Experimental Evidence. Neurosci. Biobehav. Rev..

[52] Wansink B., Cheney M., Chan N. (2003). Exploring Comfort Food Preferences across Age and Gender. Physiol. Behav..

[53] Oliver G., Wardle J., Gibson E.L. (2000). Stress and Food Choice: A Laboratory Study. Psychosom. Med..

[54] Monteiro C.A., Cannon G., Levy R.B., Moubarac J.-C., Louzada M.L., Rauber F., Khandpur N., Cediel G., Neri D., Martinez-Steele E. (2019). Ultra-Processed Foods: What They Are and How to Identify Them. Public Health Nutr..

[55] Fardet A. (2016). Minimally Processed Foods Are More Satiating and Less Hyperglycemic than Ultra-Processed Foods: A Preliminary Study with 98 Ready-to-Eat Foods. Food Funct..

[56] Herieka M., Erridge C. (2014). High-Fat Meal Induced Postprandial Inflammation. Mol. Nutr. Food Res..

[57] Hall K.D., Ayuketah A., Brychta R., Cai H., Cassimatis T., Chen K.Y., Chung S.T., Costa E., Courville A., Darcey V. (2019). Ultra-Processed Diets Cause Excess Calorie Intake and Weight Gain: An Inpatient Randomized Controlled Trial of Ad Libitum Food Intake. Cell Metab..

[58] van de Wouw M., Schellekens H., Dinan T.G., Cryan J.F. (2017). Microbiota-Gut-Brain Axis: Modulator of Host Metabolism and Appetite. J. Nutr..

[59] Giovannucci E., Taylor C.A., Deierlein A.L., Raynor H.A., Hoelscher D.M., Anderson C.A.M., Booth S.L., Fung T.T., Gardner C.D., Stanford F.C. (2024). Sugar-Sweetened Beverages and Risk of Type 2 Diabetes: A Systematic Review.

[60] Srour B., Fezeu L.K., Kesse-Guyot E., Allès B., Méjean C., Andrianasolo R.M., Chazelas E., Deschasaux M., Hercberg S., Galan P. (2019). Ultra-Processed Food Intake and Risk of Cardiovascular Disease: Prospective Cohort Study (NutriNet-Santé). BMJ.

[61] de Araújo T.P., de Moraes M.M., Magalhães V., Afonso C., Santos C., Rodrigues S.S.P. (2021). Ultra-Processed Food Availability and Noncommunicable Diseases: A Systematic Review. Int. J. Environ. Res. Public Health.

[62] Pagliai G., Dinu M., Madarena M.P., Bonaccio M., Iacoviello L., Sofi F. (2021). Consumption of Ultra-Processed Foods and Health Status: A Systematic Review and Meta-Analysis. Br. J. Nutr..

[63] Pecoraro N., Reyes F., Gomez F., Bhargava A., Dallman M.F. (2004). Chronic Stress Promotes Palatable Feeding, Which Reduces Signs of Stress: Feedforward and Feedback Effects of Chronic Stress. Endocrinology.

[64] Klatzkin R.R., Nolan L.J., Kissileff H.R. (2022). Self-Reported Emotional Eaters Consume More Food under Stress If They Experience Heightened Stress Reactivity and Emotional Relief from Stress upon Eating. Physiol. Behav..

[65] Tsenkova V., Boylan J.M., Ryff C. (2013). Stress Eating and Health: Findings from MIDUS, a National Study of U.S. Adults. Appetite.

[66] Dallman M.F., Pecoraro N., Akana S.F., La Fleur S.E., Gomez F., Houshyar H., Bell M.E., Bhatnagar S., Laugero K.D., Manalo S. (2003). Chronic Stress and Obesity: A New View of “Comfort Food”. Proc. Natl. Acad. Sci. USA.

[67] Wen J., Inauen J., Miao M. (2025). Negative Affect and Emotional Eating: Daily Dynamics and Their Links to Difficulties in Emotional Regulation. Appetite.

[68] Frayn M., Livshits S., Knäuper B. (2018). Emotional Eating and Weight Regulation: A Qualitative Study of Compensatory Behaviors and Concerns. J. Eat. Disord..

[69] Baethge C., Goldbeck-Wood S., Mertens S. (2019). SANRA—A Scale for the Quality Assessment of Narrative Review Articles. Res. Integr. Peer Rev..

[70] Manchón J., Quiles M.J., Quiles Y., López-Roig S. (2021). Positive and Negative Emotional Eating Are Not the Same—The Spanish Version of the Positive-Negative Emotional Eating Scale (PNEES). Front. Psychol..

[71] Maugeri A., Barchitta M. (2019). A Systematic Review of Ecological Momentary Assessment of Diet: Implications and Perspectives for Nutritional Epidemiology. Nutrients.

